# Flea-Borne Typhus as a COVID-19 Mimic: A Report of Four Cases

**DOI:** 10.1155/2024/9914306

**Published:** 2024-02-14

**Authors:** Bradley V. Dye, Jose Alejandro Coba, Christopher L. Dayton, Jose Cadena, Gregory M. Anstead

**Affiliations:** ^1^Department of Medicine, University of Texas Health San Antonio, 7703 Floyd Curl Drive, San Antonio, TX 78229, USA; ^2^San Antonio Infectious Diseases Consultants, 8042 Wurzbach Road, San Antonio, TX 78229, USA; ^3^Division of Pulmonary Diseases and Critical Care, Department of Medicine, University of Texas Health San Antonio, 7703 Floyd Curl Drive, San Antonio, TX 78229, USA; ^4^Department of Emergency Medicine, University of Texas Health San Antonio, 7703 Floyd Curl Drive, San Antonio, TX 78229, USA; ^5^Division of Infectious Diseases, Department of Medicine, University of Texas Health San Antonio, 7703 Floyd Curl Drive, San Antonio, TX 78229, USA; ^6^Division of Infectious Diseases, Medical Service, South Texas Veterans Health Care System, San Antonio, TX 78229, USA

## Abstract

Flea-borne typhus (FBT), due to *Rickettsia typhi* and *R. felis*, is an infection causing fever, headache, rash, hepatitis, thrombocytopenia, and diverse organ manifestations. Cough occurs in about 30% of patients with FBT, and chest X-ray abnormalities are seen in 17%. Severe pulmonary manifestations have also been reported in FBT, including adult respiratory distress syndrome and pulmonary embolism. Because of these pulmonary manifestations, FBT can mimic Coronavirus Illness 2019 (COVID-19), a febrile illness with prominent respiratory involvement. Flea-borne typhus and COVID-19 may also have similar laboratory abnormalities, including elevated ferritin, C-reactive protein, and D-dimer. However, elevated transaminase levels, rash, and thrombocytopenia are more common in FBT. Herein, we present four cases of patients with FBT who were initially suspected to have COVID-19. These cases illustrate the problem of availability bias, in which the clinician thinks a particular common condition (COVID-19 in this case) is more prevalent than it actually is.

## 1. Introduction

Flea-borne typhus (FBT), caused by the bacteria *Rickettsia typhi* and *R. felis,* is transmitted to humans by a flea bite or by the inoculation of a bite site, a skin abrasion, or mucous membranes with flea feces infected with these rickettsiae [1]. The pathologic effects of FBT are due to systemic vascular endothelial injury. This results in a wide range of variably penetrant symptoms due to multiorgan involvement, producing a spectrum of disease ranging from a self-resolving nonspecific febrile illness to organ failure and death.

In the last decade, the incidence of FBT has increased in both Texas and California [2].

Texas registered 3750 FBT cases from 2010–2019 [3]. California reported 1319 cases from 2014–2023 [4], and Hawaii had 75 cases during 2010–2019 [5]. Outside of the United States, FBT is reemerging in diverse locations worldwide. It is also recognized by returning travelers [6] and is considered a neglected tropical disease due to its occurrence in impoverished populations who are exposed to rats and stray domestic animals [7].

The COVID-19 pandemic originated in the Wuhan province of China; cases of atypical pneumonia were initially reported in December 2019, and it subsequently spread globally. As of November 2023, there have been over 770 million cases and almost 7 million deaths due to COVID-19. Thus, this infection has affected society and the practice of medicine like no other infection since pandemic influenza in 1918 [8].

COVID-19 is caused by the severe acute respiratory syndrome coronavirus 2 (SARS-CoV-2). Transmission of the SARS-CoV-2 is predominantly through droplets and short-range airborne aerosols, and the disease typically occurs after an incubation period of about 4–7 days. Clinical features usually include upper and lower respiratory tract symptoms, sometimes with anosmia and/or dysgeusia. The onset may be gradual and may include nonspecific features, including rash, fever, fatigue, arthralgias, myalgias, hepatitis, leukopenia, rhabdomyolysis, renal dysfunction, encephalitis, and myocardial infarction, among others [9]. This nonspecific presentation may cause confusion with rickettsioses, such as FBT [10].

A histopathological similarity between rickettsial illness and COVID-19 is the presence of endothelial dysfunction and vasculopathy. SARS-CoV-2 has the capacity to infect endothelial cells and cause endotheliitis, marked by viral inclusions and inflammatory infiltrates. A cytokine storm may occur during COVID-19 infection after an initial phase of high viral replication, complicating its clinical course. Fever, rash, and vascular dysfunction are also common in rickettsioses, potentially leading to multiorgan failure and death [10].

Clinically, COVID-19 may also have cutaneous manifestations, such as a truncal maculopapular rash that spares the face, palms, and soles. Petechial rashes, the Adult Respiratory Distress Syndrome (ARDS), disseminated intravascular coagulation (DIC), and acral ischemia can occur with both COVID-19 and rickettsial infections [10]. Thus, it is important to consider rickettsial infection among patients with compatible clinical features during the COVID-19 pandemic because confusion between entities may result in delayed effective therapy. The purpose of this paper is to present the cases of four patients who presented with signs and symptoms initially suspected to be COVID-19 and were found to have FBT.

## 2. Case 1

The first patient is a 30-year-old woman with no significant past medical history who presented to the emergency department in June of 2020 with a sudden onset of fever 7 days prior. She reported temperatures as high as 39.6°C, accompanied by chills. She also experienced dyspnea at rest which was worse with exertion. She took multiple doses of ibuprofen with no effect on the fevers. One day prior to presentation, she developed a bilateral temporal headache and left-sided chest and flank pain which prompted her to come to the emergency department. She denied coughing, nausea, vomiting, diarrhea, or altered sense of taste or smell. She lived in San Antonio, Texas, with both parents and two nephews all of whom were asymptomatic. She denied recent outdoor activities or attending any large gatherings and was working remotely from home. Initial vital signs were a temperature of 39.1°C, a pulse 124 beats/min, respiratory rate of 21 breaths/min, a blood pressure of 128/72 mm Hg, and an oxygen saturation of 98% on 2 L of oxygen via nasal cannula. A physical exam found clear lung sounds bilaterally, a few erythematous pustules on her left ankle and mild diffuse abdominal tenderness. No organomegaly was noted. A computerized tomograph (CT) of the chest showed peribronchovascular mixed consolidative and ground glass opacities along the lingula. Based on the patient's fever, dyspnea, and CT findings, COVID-19 was suspected. The patient's initial COVID-19 test was negative (Hologic SARS-CoV2 TMA Assay; Marlborough, MA), and a repeat test 18 hours later was also negative. Initial laboratory findings were significant for lymphopenia, thrombocytopenia, mild normocytic anemia, and elevated transaminase, troponin I, and C-reactive protein levels. Two sets of blood cultures were negative. A nasopharyngeal respiratory pathogen PCR panel (Biofire RP2.1, Salt Lake City, UT; detects 18 viral including SARS-CoV-2, and three bacterial targets) was negative. The patient was given intravenous ceftriaxone and oral azithromycin to treat community-acquired pneumonia with the patient remaining febrile for more than 24 hours on this regimen. With the continued fever, the infectious diseases service was consulted, and they found that she owned three dogs and reported that all three had acquired fleas, which she was treating with antiflea shampoo. She also reported multiple stray cats in the vicinity of her house but denied contact with them. The infectious diseases consultants recommended switching azithromycin to empiric doxycycline due to suspicion of FBT. Her fever improved with the change to doxycycline prior to discharge 3 days later. IgM and IgG *R. typhi* titers returned strongly positive at 1 : 1024 and 1 : 256, respectively. These results confirmed the diagnosis of FBT with no further need for convalescent sera.

## 3. Case 2

A 45-year-old male with a past medical history of anxiety, depression, tobacco use, and obstructive sleep apnea presented in the late spring of 2020 with 8 days of fever and a more recent onset of respiratory and neurologic symptoms. He reported that his temperatures at home were as high as 40°C. A few days prior to presentation, he was evaluated in an urgent care clinic and was prescribed a five-day course of azithromycin with no improvement in the fever. He also reported mild dyspnea, a dry cough, and wheezing. Other complaints included nausea, severe frontal headaches, mild memory loss (reported by his spouse), and vertigo. He lived in San Antonio (TX) with his wife who was currently working as a nurse in a COVID-19 unit. He also owned seven dogs with known flea infestations. Initial vital signs were a temperature of 37.6°C, a pulse 98 beats/min, a respiration rate of 18 breaths/min, a blood pressure of 146/87 mm Hg, and an oxygen saturation of 97% on room air. While in the emergency department, the patient developed a fever up to 39.5°C with rigors. On the physical exam, he appeared ill and uncomfortable, but his breath sounds were clear. A CT of the chest showed ground glass opacities with small areas of consolidation within the left lower lobe. Magnetic resonance imaging of the brain was normal. The patient's initial COVID-19 test was negative (Cepheid Xpert, real-time polymerase chain reaction (RT-PCR); Sunnyvale, CA), but due to his respiratory symptoms, abnormal CT findings, and high probability of exposure, he was admitted to the COVID-19 unit as a person under investigation. COVID-19 was subsequently ruled out 24 hours later by a second negative PCR test. Other initial laboratory findings were remarkable for hyponatremia, hyperferritinemia, and elevated transaminases, lactate dehydrogenase, D-dimer, and C-reactive protein levels. Levels of CD4, CD8, CD3, CD19, and CD16+CD56 lymphocytes were within normal limits (see Table 1). A nasopharyngeal respiratory pathogen PCR panel (Biofire RP2.1) was also negative. The patient was given an intravenous dose of 2 g of ceftriaxone for initial concerns of community-acquired pneumonia. Suspicion for flea-borne typhus was established early in the hospital course with appropriate serologies and doxycycline ordered on hospital day 1. Due to a persistent severe headache and fever, a lumbar puncture was performed on hospital day 4 with cerebrospinal fluid studies not concerning meningitis or encephalitis. The patient was discharged on hospital day 5 to complete a 7-day course of doxycycline. The initial rickettsial serologic panel returned negative for flea-borne typhus, but a convalescent rickettsial panel obtained nine weeks later demonstrated high *R. typhi* IgM and IgG titers (both >1 : 256) meeting diagnostic criteria for FBT.

## 4. Case 3

The third patient is a 62-year-old man with a past medical history of hyperlipidemia and prediabetes who presented to the emergency department in the late spring of 2021 with a 7-day history of fever (up to 39.4°C at home), rigors, night sweats, mild cough, and myalgias. He reported no improvement in flu-like symptoms with antipyretics. He also reported loss of taste and smell, an occasional nonproductive cough, nausea, anorexia, and dark urine despite increased fluid intake. He denied any shortness of breath, dyspnea on exertion, or chest pain. The patient had received two doses of an mRNA SARS-CoV-2 vaccine (Pfizer/Bio-N-Tech), with the second dose administered 6 weeks prior to presentation. Social history was significant for exposure to stray cats and dogs at his residence with reported flea bites a few weeks prior to presentation. Initial vital signs were a temperature of 37.8°C, a pulse of 125 beats/min, blood pressure of 122/81 mm Hg, and a respiratory rate 20 breaths/min, with pulse oximetry showing 97% saturation on room air. Initial physical exam was remarkable for overt shivering and clear breath sounds bilaterally. The initial laboratory results were notable for hyperferritinemia, highly elevated D-dimer, thrombocytopenia, lymphopenia (absolute lymphocyte count <300/*μ*L), hyponatremia, proteinuria (>500 mg/dL), and elevated procalcitonin, lactate dehydrogenase, total bilirubin, and transaminase levels. A CT of the chest showed a left upper lobe consolidative opacity with a small pleural effusion. A Doppler ultrasound of the extremities revealed no deep vein thrombi. SARS-CoV2 PCR testing (FluVid; detects influenza A/B, SARS-CoV2, and respiratory syncytial virus A/B; Cepheid, Sunnyvale, CA) was negative despite high clinical suspicion for COVID-19. A subsequent nasopharyngeal respiratory pathogen PCR panel (Biofire RP2.1) was also negative. Fluid administration and broad-spectrum coverage with IV ceftriaxone and oral doxycycline were initiated to empirically treat sepsis due to community-acquired pneumonia. Doxycycline was chosen for atypical organism coverage given the patient's exposures to stray animals and laboratory findings concerning possible FBT. He continued to meet systemic inflammatory response syndrome (SIRS) criteria and was given additional IV fluids, resulting in volume overload and acute hypoxic respiratory failure. He promptly improved with diuretic administration and was discharged on hospital day 7 to complete 10 days of oral doxycycline. He was retrospectively diagnosed with flea-borne typhus via convalescent serology studies six weeks postdischarge with *R. typhi* IgM and IgG titers >1 : 256.

## 5. Case 4

A 27-year-old male with a past medical history of migraine headaches presented to the emergency department in November of 2020 with 10 days of headache, fever (reported >38.9°C) chills, chest pain, and myalgias. His symptoms started gradually with a dull posterior headache, which radiated bilaterally to his forehead and the back of his eyes. He noted a distinct difference between the character of his presenting headache and his usual migraine symptoms, which typically consisted of unilateral pain, aura, photophobia, and nausea. His usual abortive migraine therapies were ineffective. His chest pain was nonexertional, correlating with local myalgias. He reported a significant COVID-19 exposure 14 days prior to presentation but tested negative multiple times via home-testing COVID-19 antigen kits. He denied any recent travel, outdoor exposures, or contacts with similar symptoms.

Vital signs at the time of presentation showed a temperature of 36.9°C, pulse 119 bpm, a blood pressure of 146/85 mm Hg, a respiratory rate of 20 breaths/min, and a pulse oximetry saturation of 100% on room air. Physical examination revealed an ill-appearance, clear breath sounds bilaterally, tachycardia, and absent nuchal rigidity, Kernig sign, and Brudzinski sign. Laboratory studies were significant for mildly elevated transaminases, mildly elevated international normalized ratio (INR), mild hypoalbuminemia, elevated sedimentation rate, and C-reactive protein levels, and a normal complete blood count. Given his symptoms of headache and concern for infection, a lumbar puncture was performed. Cerebrospinal fluid studies were remarkable for an elevated protein level with otherwise negative routine infectious meningitis/encephalitis studies. A rickettsial serologic panel was collected due to clinical suspicion of FBT, despite the lack of obvious flea exposure.

The patient was started on empiric antimicrobial therapy for meningitis/encephalitis with vancomycin, ceftriaxone, intravenous acyclovir, as well as empiric doxycycline, but his antimicrobial regimen was quickly narrowed to doxycycline monotherapy on hospital day 2 following unremarkable CSF findings. The patient defervesced on hospital day 3 with a complete resolution of headaches and myalgias by hospital day 4. He was discharged on oral doxycycline to complete an 8-day course. Acute phase sera returned after the patient was discharged with significantly elevated *Rickettsia typhi* titers for IgM >1 : 1024 and IgG >1 : 512, confirming the diagnosis of FBT. The laboratory and clinical findings of the four patients are summarized in Tables 1 and 2.

## 6. Discussion

The ubiquity of COVID-19 in the era of the pandemic cannot be understated; unfortunately, the resultant cognitive biases can undermine a clinician's diagnostic acumen. This bias may cause less common infectious etiologies, such as FBT, to be neglected in the initial differential diagnosis. Flea-borne typhus was considered early in the cases described above because there is awareness of this infection in our institutions due to the recent reemergence of this infection in Bexar County (TX). However, in other locales, diagnostic bias toward COVID-19 may result in delays in seeking care, longer hospitalizations, or increased patient morbidity.

The most relevant of the cognitive biases to the pandemic, as providers across all specialties have been confronted with a high burden of COVID-19 cases, is availability bias, in which the clinician thinks a particular common condition is more prevalent than it actually is [11]. This availability bias may be augmented by the shared features of COVID-19 and FBT in presenting symptoms, imaging features, and laboratory findings. In Case 4, it was the patient that displayed availability bias, because in the context of a global pandemic, with the news cycle dominated by a focus on COVID-19, he thought his fever and headache were likely due to this illness. It was only after multiple negative home antigen tests that the patient presented for medical care, and the correct diagnosis was made.

Anchoring bias, the tendency to rely too heavily on the first piece of information received in a particular situation [11], may play less of a role in mistaking FBT for COVID-19, as nucleic acid amplification assays for SARS-CoV2 are very sensitive (greater than 95%), allowing providers to effectively rule out the disease with one or two negative results at presentation. However, if the clinical suspicion for COVID-19 is high, for example, due to known COVID-19 exposures, these negative results may not be accepted as true. For example, the second patient in this case study was labeled as a person under investigation and placed in the COVID-19 unit despite a negative PCR assay.

COVID-19 has classically been described in three clinical phases [12]. The first is the viral response phase, which is consistent with a typical viral prodrome of fever, chills, myalgias, malaise, and upper respiratory symptoms. The pathogenesis is primarily driven by the direct cytopathic effects of viral replication. Some symptoms peculiar to early variants of SARS-CoV2 included loss of sense of taste or loss of sense of smell [9], which the third patient in this case series initially reported. Although the specific frequencies of these symptoms may differ according to individual SARS-CoV2 variants, a prodrome of nonspecific systemic symptoms appears to be generally conserved [13]. The second and third phases of COVID-19 involve worsening respiratory symptoms and a hyperinflammatory state, driven by an overexuberant immune response. The latter phase may be associated with severe clinical manifestations, including ARDS, cardiac distress, secondary infections, and shock, leading to multiorgan failure and a poor prognosis [12, 14]. Patients are typically afebrile or defervescing by the pulmonary and hyperinflammatory phases unless a secondary infection is present [12]. Flea-borne typhus can resemble the viral response phase of COVID-19, which may include nonspecific findings of fever, myalgias, headache, and malaise as well as dry cough. However, symptoms localizing to the upper respiratory tract, such as rhinorrhea or sore throat, are uncommon in FBT [15]. Less frequently, FBT can also resemble the pulmonary phase of COVID-19 with shortness of breath and hypoxia if pulmonary involvement is significant enough. Multiple nonspecific symptoms are typical in the presentation of FBT, though they may not commonly present together to compose a conserved syndrome [15].

One possible distinguishing feature of FBT is rash, which occurs in 47.6% of adult patients [15]. The rash usually starts on day five of the illness but may be missed initially because it may be high in the axillae or the inner surface of the arms. The onset of the rash may also be coincident with the fever or appear as late as 8-days after the onset of fever. The rash primarily involves the trunk, arms, and thighs [16]. In one series of 35 FBT patients with rash, it was macular (49% of cases), maculopapular (29%), papular (14%), morbilliform (3%) or petechial (6%) at the time of presentation [17]. It may be difficult to recognize the rash of FBT in individuals of darker complexions as well as its different stages of evolution during the clinical course [18]. Clinicians should perform a thorough skin examination on patients with high suspicion of FBT, as the presence of the characteristic rash can lend some specificity toward a diagnosis of FBT.

Dermatologic manifestations are less common in COVID-19 than in FBT, occurring in about 20% of cases [19]. The cutaneous lesions of COVID-19 have been classified into five patterns [20]: (1) maculopapular eruptions (47% of cases); (2) urticarial lesions (19%; truncal or dispersed); (3) acral erythema with vesicles or pustules (19%; pseudochilblains); (4) other vesicular eruptions (9%; mostly monomorphic but may be hemorrhagic); and (5) livedo or necrosis (6%; due to vaso-occlusive disease). Vesicular eruptions occur early in COVID-19, whereas pseudochilblains usually appear late; other rash patterns are concurrent with other COVID-19 manifestations [20]. Thus, the cutaneous findings of COVID-19 are distinct from those of FBT, helping to differentiate these two conditions. Digital ischemia is very rare in FBT [21].

Although FBT is usually not considered to have pulmonary manifestations, cough was reported in the Tsioutis series in 28% of patients [15]. Van der Vaart and coworkers estimate FBT causes coughs in about 30% of patients, and chest X-ray abnormalities are seen in 17% [22]. Severe pulmonary manifestations have also been reported in FBT, including ARDS and pulmonary embolism [22–25]. There is also substantial overlap in the spectrum of chest imaging findings between the two infections, from an absence of infiltrates to ground glass opacities on computed tomography to extensive infiltrates consistent with ARDS [22, 26, 27]. Pulmonary findings in FBT portend a worse prognosis [28]. The laboratory findings of the patients in the case series above are typical for FBT. Elevated transaminase levels were observed in all the patients; hyponatremia was observed in 75%, and thrombocytopenia and hypoalbuminemia in half. Lymphocytopenia is a common presentation of COVID-19 but is seldom specifically noted for FBT [15, 28]. However, lymphocytopenia was present in two of the four patients in this series and has been described in other patients with FBT [29, 30]. The findings further illustrate the potential ambiguity in distinguishing between FBT and COVID-19.

Other laboratory findings for COVID-19 and FBT can also be similar. For FBT, elevated transaminase and lactate dehydrogenase levels and hypoalbuminemia are generally conserved with each being individually present in roughly 80% of cases [15]. In contrast, early variants of COVID-19 generally displayed a lesser frequency of transaminase elevation compared to FBT [31]. Though it should be considered that remdesivir, an antiviral commonly used to treat COVID-19, results in mildly elevated transaminase levels in greater than 10% of patients [32]. Lactate dehydrogenase was elevated in 46.2% of COVID-19 cases, according to early studies from Wuhan, China; hypoalbuminemia was present in a mean of 62.9% of COVID-19 patients [31]. Thrombocytopenia may be somewhat helpful in discerning FBT from COVID-19, as it is present in roughly 50% of FBT patients [15], compared with 12.6% of patients in early COVID-19 studies [31].

Significant D-dimer elevation can be seen with either condition. Elevated D-dimer levels have not been commonly reported in FBT [25, 29] and were observed in two of the two tested patients in this series. For COVID-19 patients, D-dimer elevation was seen in 14–42% [33]. Elevated ferritin levels were found in two patients in this series but have not been commonly reported in FBT [29, 34, 35]. However, in COVID-19 serum ferritin levels >500 *μ*g/L were observed in all severe patients on admission [36]. Li et al. detected elevated ferritin levels in 90.7% of 54 COVID-19 patients [37].

The role of availability bias in delaying the diagnosis of FBT amid the COVID-19 pandemic has been previously described. Patel reported a case from California in which a persistently febrile patient was tested twice for COVID-19 at two separate medical encounters and was given empiric ceftriaxone and azithromycin, despite high neutrophilia, left shift, and lymphocytopenia [30]. Finally, at the fifth medical encounter, a more thorough history deduced that the patient was a dog trainer. After consultation with an infectious diseases specialist, serologic testing for FBT was performed, which was positive. Flea-borne typhus has also been mistaken for multisystem inflammatory syndrome in children (MIS-C), a manifestation of COVID-19 in the pediatric population, which presents as fever, elevated inflammatory markers, multiorgan involvement, and myocardial dysfunction [29, 38]. Bhatt and coworkers have also described how the overlap in presentation of COVID-19 with other infections has resulted in a significant delay in diagnoses and management of acute febrile illness in developing countries [39].

The introduction of the tetracyclines into clinical practice in 1948 revolutionized the treatment of rickettsial infections [40]. Since its approval in 1967, doxycycline has been the drug of choice for FBT (100 mg orally, twice daily). During treatment of FBT with doxycycline, the fever typically remits within 48–72 hours [41, 42], although infrequently delayed defervescence may occur [43]. Other antibiotics known to be effective against intracellular pathogens, such as fluoroquinolones and macrolides, have also been used to treat FBT. However, ciprofloxacin failures have been reported [44]. Azithromycin has been primarily used to treat FBT during pregnancy [45], but a recent clinical trial clearly demonstrated the superiority of doxycycline over azithromycin in the treatment of FBT in terms of time to fever clearance and the frequency of treatment failure [46]. In the current study, case patient #2 failed to improve while on azithromycin. Otherwise, once FBT was suspected or proven in the four patients, they received doxycycline and had rapid improvement.

## 7. Conclusions

In this paper, we have presented four cases of patients that illustrate that the signs, symptoms, radiographic findings, and laboratory results of FBT can mimic COVID-19 and that clinicians and patients may exhibit cognitive bias toward a COVID-19 diagnosis when such patients present for medical care. The limitations of the current study are that only four patients were found by passive surveillance in two hospitals. Also, for two of the laboratory tests, ferritin and D-dimer, we only had the data for two of the patients.

Cognitive biases have plagued the proper diagnosis of FBT long before the COVID-19 pandemic. Flea-borne typhus can present nonspecifically enough to be confused with a host of other infections [17]. Considering its history and epidemiology, FBT has circulated in endemic areas for decades without proper recognition owing to the limitations of diagnostic testing. Because serological methods are still the gold standard of detection, the diagnosis is commonly retrospective, often coming after a patient has been discharged following an expensive and unnecessary diagnostic evaluation. In FBT, antirickettsial antibodies are present in less than 20% of patients at seven days of illness [47], which leaves the convalescent phase sera to confirm the diagnosis much later. More expedient diagnostic modalities are needed to prevent unnecessary testing, delays in definitive treatment, and possibly harm to patients. Unfortunately, traditional nucleic acid amplification methodologies have not shown much promise, as they are insufficiently sensitive for the diagnosis of FBT [48]. Recently, next-generation sequencing techniques performed on blood have shown potential for the rapid diagnosis of FBT without the pitfalls of serologic testing [49].

It must be emphasized that a full social history is crucial to establishing or prioritizing FBT in the differential diagnosis. Though there is some merit in distinguishing the clinical and laboratory differences between COVID-19 and FBT, a careful social history can quickly bolster a working diagnosis. Furthermore, a patient's COVID-19 vaccination status will certainly complicate the interpretation of clinical and laboratory findings, as much of the existing COVID-19 literature is based on earlier variants prior to the era of mass vaccination. Although the final patient in this series presented in the fall months with no known exposure to fleas, FBT incidence in Texas is known to peak in the late spring or early summer, coinciding with the period of maximum flea activity. Clinicians should ask about exposures to domestic animals, stray or wild animals, fleas, flea bites, and outdoor exposures [50]. Obtaining a travel history is also important because FBT is endemic to specific geographic areas [6].

## Figures and Tables

**Table 1:** Initial laboratory values for patients 1–4.

Analyte	Pt 1	Pt 2	Pt 3	Pt 4	Reference range	Units
Sodium	134	132	132	138	136–145	mmol/L
Potassium	3.2	4.3	3.8	4.0	3.5–5.1	mmol/L
Creatinine	0.60	0.8	1.0	0.91	0.7–1.3	mg/dL
Albumin	3.0	4.0	3.9	3.4	3.5–5.7	g/dL
Aspartate aminotransferase	88	308	113	40	13–39	IU/L
Alanine aminotransferase	94	378	261	83	7–52	IU/L
Total bilirubin	0.9	0.4	2.4	0.4	0.3–1.0	mg/dL
Urine protein	n.d.	30	>500	0	0	mg/dL
C-reactive protein	147.8	45.6	121.1 (day 3)	83.6	<10.0	mg/L
Procalcitonin	0.32	0.62	3.63	n.d.	<0.5	ng/mL
Ferritin	n.d.	>1500	>1500	n.d.	10–322	ng/mL
D-dimer	n.d.	988	11,982	n.d.	0–230	ng/mL
White blood cell count	8.88	8.2	6.2	8.07	4–10	10^3^/*μ*L
Hemoglobin	11.1	14.7	14.8	14.0	12.8–17.1	g/dL
Platelets	104	270	134	282	150–400	10^3^/*μ*L
Lactate dehydrogenase	n.d.	553	438	n.d.	140–271	IU/L
Absolute lymphocyte count	440	1900	300	2020	900–3100	cells/*μ*L
CD4	n.d.	1046	n.d.	n.d.	323–1546	cells/*μ*L
CD8	n.d.	534	n.d.	n.d.	170–1154	cells/*μ*L
CD3	n.d.	1651	n.d.	n.d.	452–2943	cells/*μ*L
CD19	n.d.	240	n.d.	n.d.	35–519	cells/*μ*L
CD16 + CD56	n.d.	193	n.d.	n.d.	44–451	cells/*μ*L

n.d., not determined.

**Table 2:** Demographics, clinical findings, risk factors, and treatment of patients 1–4.

Clinical findings/treatment	Pt 1	Pt 2	Pt 3	Pt 4
Age (yrs), sex	30, F	45, M	62, M	27, M
Fever	Yes	Yes	Yes	Yes
Chills	Yes	No	Yes	Yes
Dyspnea	Yes	Yes	Yes	No
Headache	Yes	Yes	No	Yes
Cough	No	Yes	Yes	No
Gastrointestinal symptoms	No	Nausea	Nausea, anorexia	Nausea
Duration of illness prior to hospital presentation, days	7	8	7	10
CT of the chest	Peribroncho-vascular mixed consolidative and ground glass opacities along the lingula	Ground glass opacities with small areas of consolidation within the left lower lobe	Left upper lobe consolidative opacity with a small pleural effusion	Not performed
FBT risk factor	Dogs with fleas	Dogs with fleas	Stray cats and dogs; flea bites	None identified
Treatment	Ceftriaxone and azithromycin, then doxycycline	Azithromycin for 2-days without improvement, then doxycycline	Ceftriaxone and doxycycline	Vancomycin, ceftriaxone, acyclovir, doxycycline; narrowed to doxycycline on d3

## Data Availability

Data are available upon request to the corresponding author.
